# Influence of geographical location on the distribution of heavy metals in dairy cattle feeds sourced from two South African provinces

**DOI:** 10.1002/fsn3.4082

**Published:** 2024-03-07

**Authors:** Oluwasola Abayomi Adelusi, Oluwaseun Mary Oladeji, Sefater Gbashi, Patrick Berka Njobeh

**Affiliations:** ^1^ Department of Biotechnology and Food Technology, Faculty of Science University of Johannesburg Johannesburg South Africa; ^2^ Department of Biology and Environmental Science, Faculty of Science Sefako Makgatho Health Sciences University Pretoria South Africa

**Keywords:** dairy feed, geographical location, heavy metal, ICP‐MS, South Africa

## Abstract

The contamination of feed and food by heavy metals represents a significant concern for the health of both animals and humans. This study investigates the impact of geographical location on heavy metal distribution in dairy cattle feeds sourced from Free State and Limpopo, South Africa (SA). A total of 70 feed samples (40 from Free State and 30 from Limpopo) were collected from 2018 to 2019 and analyzed for heavy metals, including cadmium (Cd), arsenic (As), copper (Cu), zinc (Zn), lead (Pb), and chromium (Cr), using inductively coupled plasma mass spectrometry (ICP‐MS). Our findings revealed the presence of Cr, Cu, and Zn in the feeds, but at levels below the FAO/WHO permissible limits. Additionally, As, Cd, and Pb concentrations in the feeds were below the Limit of Detections (LODs). Generally, Cr concentrations (0.032–0.454 mg/kg) identified in the Free State samples were lower than those found in Limpopo (0.038–1.459 mg/kg), while the levels of Cu (0.092–4.898 mg/kg) and Zn (0.39–13.871 mg/kg) recorded in the Free State samples were higher than those from Limpopo [(0.126–3.467 mg/kg) and (0.244–13.767 mg/kg), respectively]. According to independent sample *t*‐tests, Cu and Zn levels were substantially higher (*p* ≤ .05) in Free State feeds compared to Limpopo, while Limpopo feeds exhibited significantly higher (*p* ≤ .05) Cr concentrations than Free State feeds. Despite the low recorded heavy metal levels, regular monitoring of these elements in cow diets across all SA provinces is essential for ensuring the well‐being of animals and humans.

## Graphical abstract

Heavy metal. Dairy feed. Geographical location.

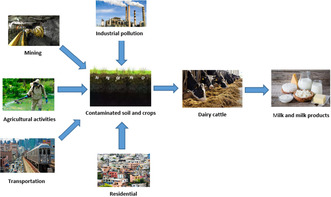

## 1 INTRODUCTION

Dairy cattle feeds are diets given to milk‐producing cows to provide them with the required nutrients for milk production. These feeds are often classified into two groups (forages and concentrates) based on their content. Forages are bulky feeds high in crude fiber that promote ruminal digestion (Govil et al., 2017), with examples ranging from dried, fresh, or ensiled forages derived from maize stalks, lucerne, and grasses, as well as by‐product feeds. Conversely, concentrates are low‐fiber, high‐energy, and highly pleasant diets. Concentrates can be high in energy (energy concentrates), such as cereals (barley, corn, wheat, and sorghum) and milling by‐products, or high in proteins (protein concentrates), such as oilseed cakes, fish meal, and sorghum. Lima et al. (2011) revealed that concentrates are more notorious than natural forages of the same quantity due to their faster fermentation in the rumen. This makes them an essential dairy diet for improving milk production. The combination of forages and concentrates produces a total mixed ration (TMR), which is needed to satisfy the dietary requirements of milk‐producing cows. It is important to note that dairy feed is a vital link in the food supply chain; the quality of dairy feeds can affect milk production, quality, and overall dairy animal health (Adelusi et al., 2022). Despite increased milk consumption in South Africa (SA) as the basis for active and healthy living, milk and milk product insecurity persists due to the carryover of various contaminants, particularly heavy metals, from feeds to dairy cattle and, subsequently, to raw milk and other dairy products.

Heavy metals are a group of pollutants that are detrimental to human and animal health. Some of these elements, such as manganese (Mn), copper (Cu), zinc (Zn), nickel (Ni), and iron (Fe), are essential to human and animal health but are toxic at higher concentrations (Noor et al., 2023; Verma et al., 2023), whereas others, such as cadmium (Cd), mercury (Hg), lead (Pb), and arsenic (As), are highly dangerous even at trace levels (Castro‐Bedrinana et al., 2021; Strumylaite et al., 2022; Verma et al., 2023). These contaminants can enter the environment via both anthropogenic and natural routes. Anthropogenic sources of heavy metal contamination include agricultural activities such as the application of fungicides, pesticides, herbicides, fertilizers, and contaminated irrigation water (Abdel‐Rahman et al., 2019; Danish & Chan, 2016; Onakpa et al., 2018; Xu et al., 2023). Additional anthropogenic sources of heavy metals include mining activities (El‐Kady & Abdel‐Wahhab, 2018; Okereafor et al., 2020), traffic emissions, as well as metallurgy and smelting (Onakpa et al., 2018). More so, non‐essential heavy metals like Cd and Pb may contaminate animal diets during feed processing (Dai et al., 2016).

Ingestion of heavy metal‐contaminated feeds and feed ingredients by dairy animals may result in their accumulation in the tissues (Petukhova, 2013), organs (Akele et al., 2022; Hashem et al., 2022), and body fluids (Hassan et al., 2019; Tahir et al., 2017) of these animals, thereby providing a primary route for human exposure via the consumption of meat (Yakupa et al., 2018), raw milk (Jaafarzadeh et al., 2023; Tahir et al., 2017), and other dairy products (Sarsembayeva et al., 2020; Sujka et al., 2019). Elevated concentrations of trace elements in feeds intended for dairy cattle have emerged as a worldwide issue, raising alarms about the potential risks they pose to the health of both animals and humans. Excessive exposure or intake of heavy metals can result in hemoglobinuria, diarrhea, gastrointestinal disorders, vomiting, tremors, ataxia, paralysis, and pneumonia (Jaishankar et al., 2014; Onakpa et al., 2018). Heavy metal poisoning in humans can impair the central nervous system and mental function and cause damage to the liver, kidney, and lungs, as well as blood composition (Amirah et al., 2013; Porova et al., 2014). Additionally, long‐term exposure may cause slowly progressing muscular, physical, as well as Alzheimer's disease, Parkinson's disease, muscular dystrophy, and multiple sclerosis (Amirah et al., 2013; Bakulski et al., 2020).

Heavy metal contamination of dairy feeds and feedstuffs has recently increased worldwide (Diyabalanage et al., 2021; Hashem et al., 2022; Hashemi, 2020; Li et al., 2019; Płaza et al., 2019), and in Africa (Abah et al., 2017; Boudebbouz et al., 2023; Diab et al., 2020). More so, previous studies conducted across SA have shown that river and bottled water (Moyo & Rapatsa, 2019; Olowoyo, Chiliza, et al., 2022), soil (Olowoyo, Lion, et al., 2022), medicinal plants (Street et al., 2008), rooibos tea (Areo & Njobeh, 2021), spices (Oladeji et al., 2023), foodstuffs (Gupta et al., 2018; Nuapia et al., 2018), fish and seafood (Bosch et al., 2017; Debipersadh et al., 2018), meat (Nuapia et al., 2018), and raw cow milk (Ataro et al., 2008) are not free from trace elements.

It has been documented that several factors, particularly geographical differences, influence the distribution and level of heavy metals in agricultural soils and plants (Afonne & Ifediba, 2020; Akenga et al., 2017; Onakpa et al., 2018; Rodríguez et al., 2022). Geographical location refers to the specific position of a piece of land or a farming area on the Earth's surface. This includes the latitude, longitude, and elevation of the location. Location refers to the specific combination of climate, topography, and soil characteristics that define a particular agricultural area. These factors play a role in the behavior and fate of heavy metals in the soil, affecting their availability for uptake by plants and subsequent transfer into the food chain (Afonne & Ifediba, 2020). Thus, understanding the impact of geographical location on heavy metal distribution is vital for food and feed safety. Despite several reports on the incidence and health risks of trace elements in SA, there is no information on the prevalence of these toxicants in cow feeds in the country. Furthermore, there is a lack of data on how geographical differences impact the distribution of heavy metals in dairy cattle feeds globally, including SA. Therefore, the objectives of this study were to (1) determine the prevalence of six heavy metals (Cd, Ni, Cu, Pb, Cr, and Cd) in smallholder dairy cattle feeds and feed ingredients collected from Limpopo and the Free State of SA and (2) assess the effects of geographical locations on the levels of trace elements in the feed samples. The results obtained from this study have the potential to contribute toward the development of efficient management strategies aimed at minimizing or alleviating the presence of harmful trace elements in dairy cow diets and feed ingredients, as well as their subsequent transfer to milk, meat, and other dairy products, not only in the studied areas but also in other African countries.

## 2 MATERIALS AND METHODS

### 2.1 Study area description

**FIGURE 1:**
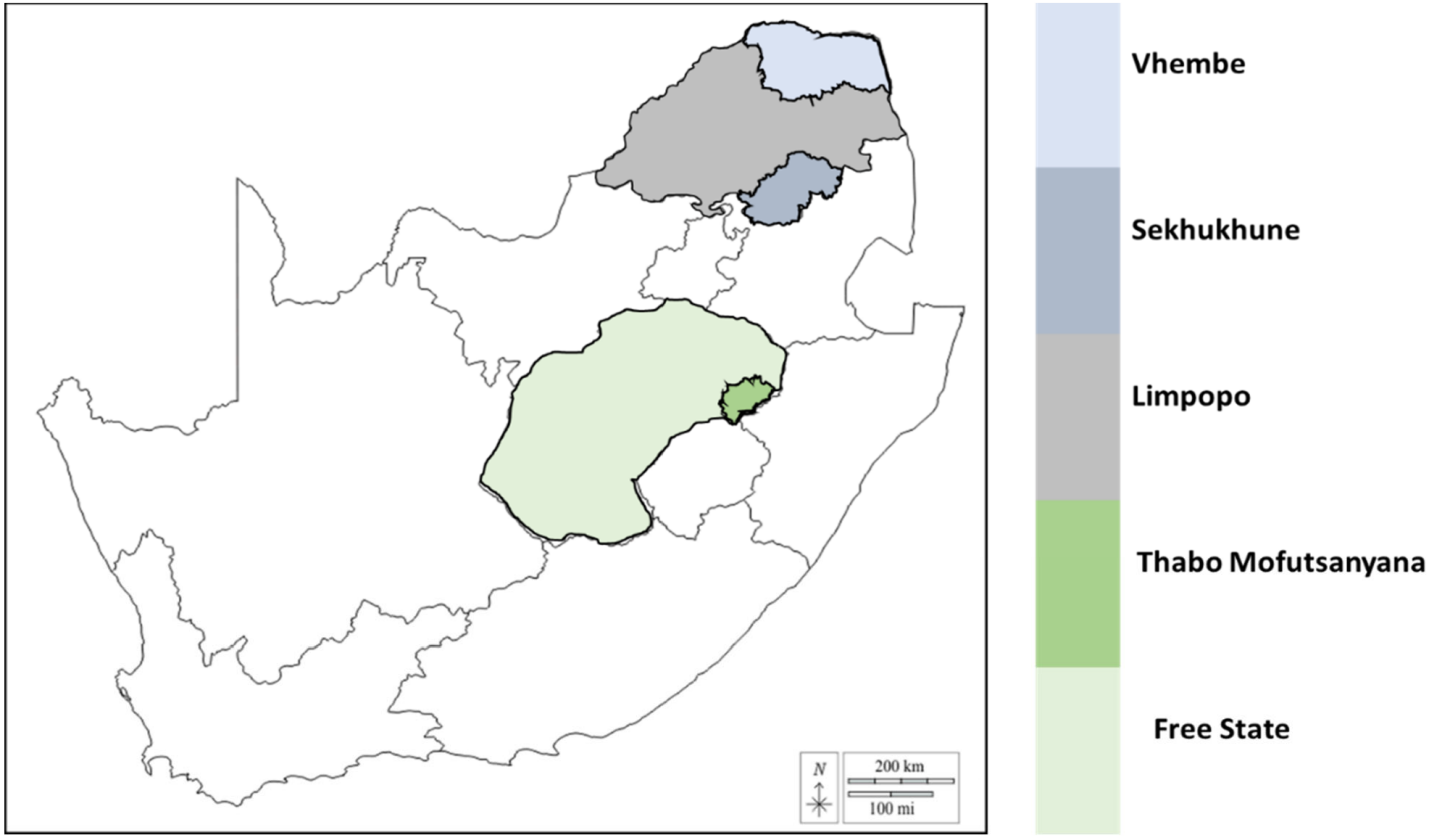
Map of SA showing the study's sampling areas.

This study was performed between 2018 and 2019 in Phutaditjaba district (Free State), with GPS coordination of *28°19′34.5″S 28°54′33.0″ E*, and Sekhukhune and Vhembe districts (Limpopo), with GPS coordination of 25°08′47.2”S29°26′17.8″ E and *23°12′35.7”S30°15′41.3″ E, respectively* (Figure 1). These areas are essential dairy‐producing regions in SA. Limpopo Province, located far north of SA, is mainly characterized by warm arid to semi‐arid or sub‐humid tropical climates. The Free State, centrally located, is primarily characterized by cooler arid to semi‐arid subtropical climates. Twenty smallholder dairy milk producers in the Phutaditjaba district (Free State) and Sekhukhune and Vhembe districts (Limpopo) were chosen for this study.

### 2.2 Sample collection

A total of 70 dairy feed samples (40 from Free State and 30 from Limpopo), including commercial feeds (*n* = 13), forages (*n* = 36), and TMR (*n* = 21), were donated by local individual participating farmers. Approximately 400 g of each feed sample was collected into sterile, plastic zip‐lock bags, kept chilled, and transported to the University of Johannesburg's Food Technology Laboratory, where they were dried, milled to fine particles, and kept frozen at −4°C before analysis.

### 2.3 Sample preparation

#### 2.3.1 Micro‐wave digestion

Approximately 0.5 g of each feed sample was placed in Teflon vessels, and subsequently, 10 mL of 65% concentrated HNO_3_ was added. The vessels were sealed using screw valves, subjected to heating at 1200 W with a microwave system, and then processed through a microwave digestion program (refer to Table 1). The resulting clear solutions were diluted to a final volume of 50 mL with deionized water and filtered through Whatman No. 1 filter paper (Whatman Ltd., England). After that, the solutions were transferred into clean polythene bottles (50 mL) for metal analysis via LCP‐MS. Likewise, a blank solution was prepared following the same procedure as the samples.

**TABLE 1:** Microwave oven program.

No	Temperature (°C)	Status	Time (min)
1	200	Ramp	20
2	200	Hold	10
3	800	Cooking	20

### 2.4 Chemicals and instrumentation

Microwave digestion system CEM 5 (CEM Corporation), an ICP‐MS NexION 300Q (PerkinElmer), 65% ultra‐pure nitric acid (HNO_3_), ultra‐pure deionized water (18.2 M cm), acetone, and hydrogen peroxide (H_2_O_2_) were used throughout this study. Additionally, 99% pure argon (Afrox) was used as a protective and purging gas (Table 2).

**TABLE 2:** ICP‐MS parameters.

Instrument	ICP‐MS (NexION 300Q)
ICP RF Power	1050 W
Integral Time	36 s
Atomizer Flow	0.89 L/min
Plasma Gas Flow	16 L/min
Scanning Time	20 s
Acquisition Mode	Jump Peak
Detector	Double Mode
Repetition	3 Times
Auxiliary Gas Flow	1.2 L/Min

### 2.5 Quality assurance and control

The validation of the study's methodology included an assessment of parameters like linearity, limits of detection (LOD), limits of quantification (LOQ), and recovery. LODs were determined by multiplying the ratio of residual standard deviation to the slope by 3.3 (according to Equation 1), and LOQs were calculated by multiplying the same ratio by 10 (as per Equation 2), following the method described by Adelusi et al. (2023). Also, the recovery was calculated according to equation (3). Meanwhile, blank and triplicate samples were analyzed during the procedure.

(1) $$\mathrm{LOD}=3.3\times \frac{\text{Residual standard deviation of the regression line}}{\text{Slope}}$$

(2) $$\mathrm{LOQ}=10\times \frac{\text{Residual standard deviation of the regression line}}{\text{Slope}}$$

The recovery was evaluated by spiking the feed samples (1 g) with known amounts of standard metals (1 ppm), while the percentage recovery was calculated using the equation below:

(3) $$\%\text{Recovery}=\frac{\text{Concentration of Spiked sample}–\text{Concentration of}\;\mathrm{Un}‐\text{spiked sample}}{\text{Concentration of known Spike added}}\times 100$$

### 2.6 Statistical analysis

The IBM Statistical Package for SPSS 22.0 (SPSS®Inc.) was used for data analysis. The mean levels of heavy metals in both provinces (Limpopo and Free State) were compared using an independent sample *t*‐test with a significance level of 95% (*p* < .05).

## 3 RESULTS AND DISCUSSION

Dairy cattle feeds and feedstuffs are indispensable not only to feed producers and dairy farmers but also to processors, policymakers, regulators, and end‐product consumers. Monitoring the harmful and potentially toxic components in bovine diets is vital for maintaining the quality and safety of dairy cattle feeds, milk, and milk products. In this study, six heavy metals (Cu, Pb, Cr, Zn, As, and Cd) contaminating smallholder dairy cattle diets in the Free State and Limpopo provinces of SA were determined and quantified using ICP‐MS. The results demonstrated acceptable reproducibility for the analysis of feed samples and thus validated the procedures employed. As indicated in Table 3, the analytical technique confirmed linearity, with *R* ^2^ values ranging from 0.9960 to 1 for all heavy metal values. More so, the LODs and LOQs of various heavy metals were between 0.03 and 0.24 and 0.1 and 0.73 mg/kg, respectively, while the recovery of each heavy metal varied from 83 to 113.5%, respectively.

**TABLE 3:** Linearity, LOD, LOQ, and recovery for heavy metal determination.

Metals	_*R*_^2^	LOD	LOQ	Recovery
Cr	1	0.03	0.1	83
Cu	1	0.03	0.1	80.4
Zn	.9960	0.24	0.73	88
As	1	0.03	0.1	113.5
Cd	1	0.1	0.3	102.1
Pb	1	0.04	0.1	99.3

Abbreviations: LOD, limit of detection; LOQ, limit of quantification; *R* ^2^, linearity.

Table 4 and Table S1 present the concentrations of heavy metals found in the feed samples collected from both geographical zones (Free State and Limpopo) and the permissible limits for heavy metals set by the WHO/FAO for dairy feeds. Furthermore, the levels of trace elements recovered from the feed samples in this study, in contrast to those documented in other regions of the world, are provided in Table 5. Our results showed that none of the tested feed samples contained As, Cd, or Pb. Interestingly, our As and Cd results are comparable to those of Koc et al. (2009), who reported that the As and Cd contents of all feed ingredients (barley, wheat, and sunflower) collected in Tekirdag, Turkey, were below the detection values. Similarly, Corguinha et al. (2015) reported that the As level in Brazilian rice was below the detection limit. The findings on Cd accorded with those reported by Hashem et al. (2022), where all cow feed ingredients and commercial feeds from Bangladesh analyzed for Cd fell below the LOD. However, these findings contradict several previous reports (Table 5). The variation in Pb, Cd, and As contamination levels in the current study and earlier investigations could be attributed to the differences in climatic conditions, raw material sources, and production process conditions that vary from factory to factory and nation to nation.

**TABLE 4:** Heavy metal concentrations (mg/kg) in smallholder dairy cattle feeds and feedstuffs from the Free State and Limpopo provinces of SA, and the WHO/FAO permissible limits.

	As	Cd	Cr	Cu	Pb	Zn
Free State						
Mean	<LOD	<LOD	0.153	0.652	<LOD	2.372
±SD	<LOD	<LOD	0.103	0.792	<LOD	2.549
Min	<LOD	<LOD	0.032	0.092	<LOD	0.39
Max	<LOD	<LOD	0.454	4.898	<LOD	13.871
Limpopo						
Mean	<LOD	<LOD	0.220	0.594	<LOD	1.977
±SD	<LOD	<LOD	0.265	0.607	<LOD	2.662
Min	<LOD	<LOD	0.038	0.126	<LOD	0.244
Max	<LOD	<LOD	1.459	3.467	<LOD	13.767
WHO/FAO	0.5	0.2	5	20	0.3	60

Abbreviations: LOD, limit of detection; Max, maximum; Min, minimum; SD, standard deviation.

**TABLE 5:** Comparison of heavy metal levels (mg/kg) in dairy cattle feeds and feedstuffs from this study and other studies.

Country	As	Cd	Cr	Cu	Pb	Zn	References
South Africa	<LOD	<LOD	0.032–1.459	0.092–4.898	<LOD	0.244–13. 871	This study
Iran	–	0.001–0.076	–	0.281–24.67	0.007–0.696	0.000–113.90	Hashemi, 2020
Namibia	0.30–0.41	–	8.40–9.20	6.88–7.30	0.44–0.60	–	Abah et al., 2017
China	–	0.02–2.72	4.61–371.60	0.27–114.27	1.85–98.64	1.330–973.44	Li et al., 2019
Sri Lanka	0.002–0.213	0.007–0.27	0.489–4.11	0.0014–0.037	0.128–0.9	0.006–0.048	Diyabalanage et al., 2021
Turkey	<LOD	<LOD	–	2.21–4.50	<0.001–1.28	13.400–60.360	Koc et al., 2009
Nigeria	–	ND – 0.5	0.001–0.8	ND–8.8	ND–8.9	–	Ifie et al., 2022
China	0.01–6.12	ND –23.25	<LOD–0.05	2.73–114.68	–	11.070–346.12	Zhang et al., 2012
Bangladesh	–	<LOD	0.095–0.286	0–0.215	0.164–0.301	0.124–1.209	Hashem et al., 2022
Pakistan	–	1.1–4.1	0.013–0.071	–	2.236–5.398	–	Tahir et al., 2017
Nigeria	–	ND–1.87	–	4.01–8.78	209–899	17.400–202	Ogundiran et al., 2012
Iran	<LOD–3	<LOD–2.5	–	–	<LOD–4.9	–	Eskandari & Pakfetrat, 2014
Pakistan	–	0.22–0.55	1.1–1.37	6.5–7.3	1.16–1.46	30.1–36.1	Batool et al., 2016
China	<LOD–0.94	0.10–5.21	0.7–19.9	<LOD–45.9	0.6–14.9	10.3–378.3	Wang et al., 2013
Spain	0.011–11.731	0.002–0.148	0.06–9.99	1.07–26.47	0.005–2.225	1.8–106.9	Orjales et al., 2018

Abbreviations: <, less than; LOD, limit of detection; ND, not detected.

**FIGURE 2:**
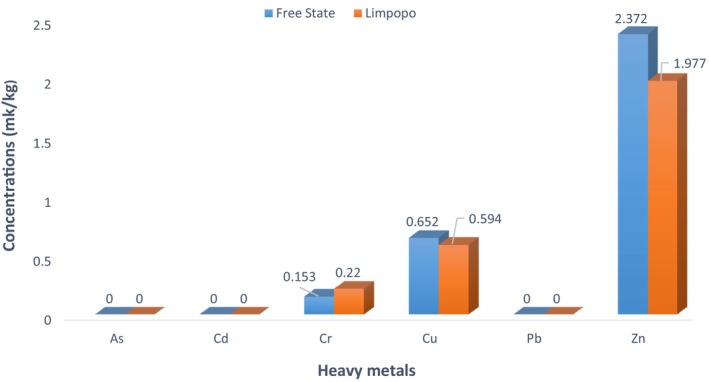
Heavy metal mean levels (mg/kg) in smallholder dairy cattle feeds and feed ingredients from the Free State and Limpopo provinces of SA.

Presently, SA has not established maximum admissible levels for Cu, Zn, and Cr in dairy feed. However, the World Health Organization (WHO) and Food and Agriculture Organization (FAO) have set maximum values of Cu, Zn, and Cr at 20, 60, and 5 mg/kg, respectively (WHO/FAO, 2007). Interestingly, Cr, the only heavy metal found at considerably higher levels in the Limpopo feed samples compared to those from Free State, was identified in all feed components, but at lower concentrations than Cu and Zn. The data presented in Table 4 provide insights into the Cr levels in dairy feeds from the two regions. The lowest Cr level (0.032 mg/kg) was observed in the Free State samples, while the highest level (1.459 mg/kg) was found in Limpopo. The Cr concentration in the Free State feeds varied from 0.032 to 0.454 mg/kg, with a mean level of 0.153 mg/kg. In contrast, the Limpopo feeds had Cr concentrations ranging from 0.038 to 1.459 mg/kg, with an average of 2.372 mg/kg. As indicated in Table 4 and Figure 2, the levels of Cr in the Limpopo feed samples were significantly (*p* < .05) higher than those in the Free State. Interestingly, the Cr levels found in this study were similar to those reported by Orjales et al. (2018) on Cr contamination in Spanish alfalfa, with amounts ranging from 0.14 and 1.30 mg/kg. However, the authors recorded higher Cr contents in other feed samples, mainly pasture, varying from 0.55 to 9.99 mg/kg, respectively. Moreover, Li et al. (2019) found extremely high amounts of Cr (4.61 to 371.60 mg/kg) in feeds fed to dairy cattle in intensive Chinese dairy farms. The Cr concentrations observed in samples from both locations in the present study were found to be lower than the permissible limit of 5 mg/kg recommended by the WHO/FAO (WHO/FAO, 2007).

The high Cr amounts observed in feed samples from Limpopo in comparison to those from the Free State are unsurprising given that SA has the world's largest Cr deposit, with the majority of Cr mines located in Limpopo (Coetzee et al., 2020), resulting in Cr pollution of the soil and feedstuffs. Another factor that could account for differences in Cr levels in the feed samples from both provinces is irrigation. Most farmers in Limpopo (the country's driest province) rely on irrigation owing to rainfall shortages. Irrigation with sewage and industrial wastewater causes heavy metal accumulation in forages and cereals worldwide (Meng et al., 2016), rendering them unfit for consumption by dairy cattle.

As revealed by Table 4, the Cu levels reported in the analyzed feed samples varied from 0.092 to 4.898 mg/kg, with the Free State feed samples containing greater amounts of Cu (mean: 0.652; range: 0.092–4.898 mg/kg) than those from Limpopo (mean: 0.594; range: 0.126–3.467 mg/kg). The amount of Cu found in the Free State feed samples was substantially (*p* < .05) higher than that collected from Limpopo (Table 6 and Figure 2). Several studies have documented minimal levels of Cu in corn silage and dairy feedstuffs, ranging from 2 to 6.4 and 2.21 to 4.50 mg/kg, respectively (Koc et al., 2009; Li et al., 2005), which are comparable to those found in this study. The Cu content (0.092–4.898 mg/kg) observed in this study was significantly lower (1.07–26.47 mg/kg) than that reported by Orjales et al. (2018) in Spanish dairy feeds. Similarly, Zhang et al. (2012) and Li et al. (2019) revealed high levels of Cu in feeds destined for milking cows in China compared with the findings of this study. In this study, Cu contents in the Free State feed samples, although higher than in Limpopo samples, did not exceed the 20 mg/kg WHO/FAO acceptable limits, making such feed safe for dairy cows.

**TABLE 6:** Independent sample *t*‐test showing variation in heavy metal mean levels (mg/kg) in smallholder dairy cow feeds and feed components from the Free State and Limpopo provinces of SA.

Heavy metal	Location	Mean + SD	DF	T	Sig
As	Free State	>LOD	>LOD	>LOD	>LOD
	Limpopo	>LOD			
Cd	Free State	>LOD	>LOD	>LOD	>LOD
	Limpopo	>LOD			
Cr	Free State	0.153 ± 0.006	4	−5.458	0.005
	Limpopo	0.220 ± 0.021			
Cu	Free State	0.652 ± 0.008	4	4.231	0.013
	Limpopo	0.594 ± 0.223			
Pb	Free State	>LOD	>LOD	>LOD	>LOD
	Limpopo	>LOD			
Zn	Free State	2.372 ± 0.044	4	10.524	0.000
	Limpopo	1.977 ± 0.048			

Abbreviations: DF, degree of freedom; LOD, limit of detection; SD, standard deviation; *T*, *t*‐value.

The differences in Cu concentrations in dairy feeds from both provinces can be linked to differences in soil factors (pH, nutrient availability, and organic matter) that control the movement of heavy metals such as Cu in soil (Afonne & Ifediba, 2020; Onakpa et al., 2018). Furthermore, Cu discrepancies in the feed samples from both areas may also be attributed to distinct agro‐climatic factors and the use of fungicides and fertilizers in agriculture. These chemicals contain high amounts of heavy metals like Cd, Pb, and Cu (Areo & Njobeh, 2021). The addition of Cu to feeds (commercial feeds) by feed producers may also account for the variation in Cu concentrations recorded in feeds from both geographical regions. As reviewed by López‐Alonso and Miranda (2020), supplementing Cu above the required levels to prevent deficiency in dairy feeds has resulted in higher Cu levels in feeds and outbreaks of Cu poisoning in dairy cattle recently. During sampling, it was observed that some dairy farmers, especially in the Free State, rely on commercial meals to feed their cattle, while others use TMR produced from commercial feeds and forages. Hence, the reason for the high Cu recorded in the Free State sample in comparison to those from Limpopo.

In this study, Zn was present in all feed components and was generally found in a higher amount than Cr and Cu. The Zn contents in the two provinces ranged from 0.244 to 13.871 mg/kg, with the Free State samples containing higher Zn levels (mean: 2.37 mg/kg; range: 0.39–13.871 mg/kg) than the Limpopo feed samples (mean: 1.977 mg/kg; range: 0.244–13.767 mg/kg) (Table 4). However, the values were below the WHO/FAO guideline of 60 mg/kg (WHO/FAO, 2007). Furthermore, Zn levels in the Free State feed samples were significantly (*p* < .05) higher than those recorded in the Limpopo feed samples (Table 6 and Figure 2). This study revealed higher Zn levels than those reported by Diyabalanage et al. (2021) in Sri Lankan dairy feed ingredients. Ogundiran et al. (2012), Li et al. (2019), and Hashem et al. (2022), on the other hand, revealed a higher level of Zn in dairy cattle feeds from Nigeria, China, and Bangladesh, respectively. The addition of fertilizers to the soil may account for the observed Zn level in the feed and feedstuffs, as nitrilotriacetic acid (NTA) fertilizer increases the accumulation of Zn in maize, an important diary feed ingredient in SA (Fässler et al., 2010). In addition, the variability in Zn levels detected in the feed samples from both geographical locations could be due to differences in farming zones and environmental conditions (Barone et al., 2016; Karimzadeh et al., 2013).

Heavy metal toxicity (poisoning) can cause an array of life‐threatening symptoms and irreversible damage. They can interfere with biological processes such as growth, differentiation, and apoptosis (Balali‐Mood et al., 2021). Research performed by Bortey‐Sam et al. (2015) to assess human health risks from trace elements through the consumption of animal‐derived food in the municipality of Tarkwa in the western region of Ghana confirmed the accumulation and distribution of various trace elements in the offal and muscle of livestock, with emphasis on the public health risks associated with the consumption of these animal by‐products. Persistence exposure to As is hazardous to humans, particularly infants and children. Indeed, arsenic (As) has been associated with reduced intelligence quotients (IQs), substandard intellectual performance, compromised cognitive function, and the occurrence of cancer in humans, as evidenced by studies such as Porova et al. (2014) and Zhou et al. (2019). Furthermore, the International Agency for Research on Cancer (IARC) has classified arsenic as carcinogenic to humans (IARC, 2014).

In contrast, Pb and Cd are non‐essential elements that adversely affect human and livestock health. A past study suggested that milk‐producing cows may be more vulnerable to the accumulation of Pb and Cd than meat‐producing cattle (Li et al., 2005). Both metals cause damage to the heart, blood vessels, immune system, and digestive tract (Zhong et al., 2016; Ziarati et al., 2018). The IARC has classified Cd and Pb as human carcinogens (Group 1) based on evidence that long‐term exposure to both trace elements is linked to an elevated risk of prostate, lung, liver, kidney, and urinary tract cancers in humans (Ziarati et al., 2018).

It is crucial to note that Cu is one of the most important metals with vitamin‐like effects in the human body (Sobhanardakani, 2018). It is required for a wide range of biological processes, such as connective tissue formation, hair pigmentation, and enzyme function (Ahuja et al., 2015). Nevertheless, an elevated intake of copper in the diet can lead to serious health complications in cattle, including damage to the liver and kidneys, methemoglobinemia, and hemolytic anemia (Dai et al., 2016; Yang et al., 2004). Similarly, Cr is a severe environmental pollutant that has received much attention due to its broad toxicity in animals and humans. The primary health risks associated with Cr include bronchial asthma, skin allergies, lung cancers, and reproductive and developmental disorders. Excessive Cr exposure can result in death (Chatterjee, 2015). Cattle are believed to be more resistant to Cu accumulation and poisoning; however, in recent years, more outbreaks of Cu toxicity in cattle have been reported globally (Bidewell et al., 2013; Suttle et al., 2013). High dietary Cu intake can cause severe health problems in cattle, including liver, hepatic, and kidney damage, methemoglobinemia, and hemolytic anemia (Dai et al., 2016; Yang et al., 2004). Zinc is a vital element required for plant, microbial, animal, and human growth and development (Chasapis et al., 2012), yet it is toxic at high levels. In humans, daily intake of 150–450 mg/kg of Zn has been linked to low Cu status, reduced immune function, altered Fe function, and reduced levels of high‐density lipoprotein (Fraga, 2005).

## 4 CONCLUSION

This study evaluated how geographic differences impact the concentrations of trace metals in feeds and feedstuffs for dairy cattle, focusing on two SA provinces (Limpopo and Free State). Despite the fact that the levels of trace elements identified in the feed samples were below the acceptable limit set by WHO/FAO for heavy metals in dairy cow diets, the notable variations observed in the amount of trace elements present in the dairy feeds from the two different geographical areas indicate the need for regular monitoring of these elements in SA cow feeds. Thus, the variations in trace element concentrations found in the two areas could be attributed to certain conditions, including agricultural practices, soil properties, agro‐climatic factors, and farm proximity to trace element mines. By investigating the influence of geographical location on the distribution of heavy metals in dairy feeds, effective strategies can be implemented to mitigate potential risks associated with heavy metal exposure to dairy cattle, as well as the transfer of these toxicants from feed to meat and other dairy products in SA and other African nations. Finally, the findings from this study could be a significant reference for mitigating heavy metal contamination in dairy feeds and, subsequently, in human diets in SA.

## AUTHOR CONTRIBUTIONS

**Oluwasola Abayomi Adelusi:** Conceptualization (lead); data curation (lead); formal analysis (equal); methodology (lead); software (equal); visualization (lead); writing – original draft (lead); writing – review and editing (equal). **Oluwaseun Mary Oladeji:** Data curation (equal); methodology (equal); validation (lead); visualization (equal). **Sefater Gbashi:** Formal analysis (equal); investigation (equal); software (lead); visualization (equal). **Patrick Berka Njobeh:** Funding acquisition (lead); project administration (lead); resources (lead); writing – review and editing (equal).

## FUNDING INFORMATION

This research was funded by the National Research Foundation (NRF), Grant number 115579.

## CONFLICT OF INTEREST STATEMENT

The authors declare that they have no conflicts of interest.

## INSTITUTIONAL REVIEW BOARD STATEMENT

This research followed the principles outlined in the Declaration of Helsinki and obtained consent from the Ethics Committee of the Faculty of Science at the University of Johannesburg (Reference number: 20160303; Approval date: March 3, 2016).

## Supporting information


**Table S1**.

## Data Availability

The raw datasets collected during this study can be made available upon request from the corresponding authors.
