# Supplementary material for: Influence of geographical location on the distribution of heavy metals in dairy cattle feeds sourced from two South African provinces
Source: Food Sci Nutr. 2024 Mar 7;12(6):4223–32. doi: 10.1002/fsn3.4082 (PMC11167146; doi:10.1002/fsn3.4082)
Supplement: Supplementary file 1 — Table S1. [file FSN3-12-4223-s001.docx]

**Supplementary Table 1**: Concentrations of heavy metals in smallholder dairy feeds and feed ingredients from the Free State and Limpopo provinces of SA.

| **Samples ID** | **Cr (mg/kg)** | **Cu (mg/kg)** | **Zn (mg/kg)** | **As (mg/kg)** | **Cd (mg/kg)** | **Pb (mg/kg** | **Feed source** |
| --- | --- | --- | --- | --- | --- | --- | --- |
| 1 | 0.171 | 0.127 | 0.759 | 0.004 | 0.000413 | 0.006862 | Limpopo |
| 2 | 0.205 | 0.961 | 2.296 | 0.003 | 0.000496 | 0.006808 | Limpopo |
| 3 | 0.156 | 0.647 | 0.506 | 0.002 | 0.000336 | 0.010611 | Limpopo |
| 4 | 0.090 | 0.149 | 0.815 | 0.000 | 0.000465 | 0.002827 | Free State |
| 5 | 0.321 | 0.325 | 2.241 | 0.020 | 0.000692 | 0.00967 | Limpopo |
| 6 | 0.087 | 0.684 | 0.588 | 0.001 | 0.000305 | 0.007348 | Limpopo |
| 7 | 0.402 | 0.275 | 0.747 | 0.005 | 0.000399 | 0.038754 | Limpopo |
| 8 | 0.100 | 1.032 | 0.548 | 0.004 | 0.000229 | 0.018031 | Free State |
| 9 | 0.360 | 0.539 | 1.040 | 0.005 | 0.000338 | 0.012817 | Free State |
| 10 | 0.039 | 0.600 | 1.206 | 0.002 | 0.000379 | 0.008441 | Free State |
| 11 | 0.043 | 0.233 | 0.979 | 0.002 | 0.000362 | 0.009359 | Free State |
| 12 | 0.127 | 1.085 | 0.247 | 0.006 | 0.000253 | 0.022831 | Limpopo |
| 13 | 0.038 | 0.445 | 0.486 | 0.003 | 0.000221 | 0.00365 | Limpopo |
| 14 | 0.077 | 0.588 | 0.759 | 0.010 | 0.000516 | 0.011104 | Limpopo |
| 15 | 0.159 | 0.260 | 0.873 | 0.009 | 0.000603 | 0.021265 | Limpopo |
| 16 | 0.046 | 1.550 | 4.749 | 0.002 | 0.00057 | 0.00481 | Free State |
| 17 | 0.161 | 0.542 | 4.719 | 0.011 | 0.001224 | 0.008584 | Limpopo |
| 18 | 0.080 | 0.832 | 0.390 | 0.003 | 0.000234 | 0.004278 | Free State |
| 19 | 0.174 | 0.087 | 0.251 | 0.001 | 0.000306 | 0.002049 | Limpopo |
| 20 | 0.076 | 1.012 | 0.872 | 0.004 | 0.000291 | 0.008408 | Limpopo |
| 21 | 0.269 | 0.126 | 0.608 | 0.001 | 0.000427 | 0.004153 | Limpopo |
| 22 | 0.204 | 0.732 | 5.197 | 0.009 | 0.001202 | 0.00639 | Limpopo |
| 23 | 0.160 | 0.246 | 0.843 | 0.001 | 0.00047 | 0.011645 | Free State |
| 24 | 0.043 | 0.290 | 1.226 | 0.002 | 0.00056 | 0.004338 | Free State |
| 25 | 0.058 | 1.390 | 2.620 | 0.002 | 0.00099 | 0.011425 | Free State |
| 26 | 0.078 | 0.328 | 1.284 | 0.001 | 0.000721 | 0.009586 | Limpopo |
| 27 | 0.185 | 0.558 | 1.427 | 0.022 | 0.000551 | 0.036624 | Limpopo |
| 28 | 0.069 | 0.558 | 0.766 | 0.002 | 0.000216 | 0.008085 | Limpopo |
| 29 | 0.430 | 0.092 | 0.612 | 0.003 | 0.000483 | 0.004325 | Free State |
| 30 | 0.180 | 1.020 | 1.921 | 0.004 | 0.000499 | 0.01013 | Free State |
| 31 | 0.151 | 0.974 | 8.098 | 0.002 | 0.000827 | 0.008184 | Free State |
| 32 | 0.298 | 0.614 | 1.492 | 0.003 | 0.000459 | 0.010962 | Free State |
| 33 | 0.213 | 0.510 | 1.529 | 0.002 | 0.000471 | 0.014093 | Free State |
| 34 | 0.104 | 0.863 | 3.290 | 0.007 | 0.001063 | 0.015096 | Free State |
| 35 | 0.032 | 0.408 | 0.745 | 0.001 | 0.000246 | 0.003938 | Free State |
| 36 | 1.495 | 0.430 | 0.876 | 0.007 | 0.000365 | 0.021895 | Limpopo |
| 37 | 0.201 | 0.568 | 4.051 | 0.002 | 0.000736 | 0.005829 | Free State |
| 38 | 0.120 | 4.898 | 13.871 | 0.002 | 0.000999 | 0.006857 | Free State |
| 39 | 0.177 | 0.098 | 0.807 | 0.003 | 0.000253 | 0.005938 | Free State |
| 40 | 0.032 | 0.150 | 0.547 | 0.000 | 0.000173 | 0.0014 | Free State |
| 41 | 0.132 | 0.298 | 2.321 | 0.005 | 0.000552 | 0.009357 | Free State |
| 42 | 0.104 | 0.417 | 1.485 | 0.002 | 0.000398 | 0.009093 | Free State |
| 43 | 0.084 | 0.434 | 1.996 | 0.003 | 0.000539 | 0.019401 | Free State |
| 44 | 0.196 | 0.397 | 2.245 | 0.003 | 0.000381 | 0.014285 | Free State |
| 45 | 0.192 | 0.268 | 1.033 | 0.002 | 0.000705 | 0.006209 | Free State |
| 46 | 0.169 | 0.715 | 6.914 | 0.003 | 0.000627 | 0.005404 | Free State |
| 47 | 0.134 | 0.566 | 4.240 | 0.008 | 0.000737 | 0.004528 | Limpopo |
| 48 | 0.176 | 1.420 | 3.569 | 0.003 | 0.001168 | 0.004192 | Free State |
| 49 | 0.246 | 0.311 | 0.883 | 0.003 | 0.000774 | 0.009836 | Free State |
| 50 | 0.082 | 0.469 | 0.448 | 0.000 | 0.000226 | 0.004324 | Limpopo |
| 51 | 0.233 | 0.308 | 3.076 | 0.004 | 0.00095 | 0.016066 | Free State |
| 52 | 0.085 | 0.112 | 0.489 | 0.001 | 0.000235 | 0.002862 | Free State |
| 53 | 0.076 | 0.158 | 1.416 | 0.003 | 0.000525 | 0.004451 | Free State |
| 54 | 0.052 | 0.256 | 0.873 | 0.002 | 0.000366 | 0.004095 | Free State |
| 55 | 0.186 | 0.789 | 1.674 | 0.021 | 0.000457 | 0.006952 | Free State |
| 56 | 0.065 | 0.676 | 2.221 | 0.015 | 0.000407 | 0.004919 | Free State |
| 57 | 0.181 | 0.153 | 0.419 | 0.001 | 0.00037 | 0.009291 | Free State |
| 58 | 0.049 | 0.350 | 0.244 | 0.006 | 0.000327 | 0.004944 | Limpopo |
| 59 | 0.164 | 0.302 | 1.997 | 0.018 | 0.000908 | 0.005946 | Limpopo |
| 60 | 0.229 | 0.592 | 3.502 | 0.008 | 0.001051 | 0.004129 | Free State |
| 61 | 0.123 | 0.661 | 4.575 | 0.008 | 0.001127 | 0.008375 | Limpopo |
| 62 | 0.018 | 0.264 | 0.395 | 0.000 | 0.000179 | 0.001367 | Free State |
| 63 | 0.454 | 0.637 | 4.303 | 0.007 | 0.001411 | 0.034148 | Free State |
| 64 | 0.301 | 0.693 | 1.506 | 0.010 | 0.000449 | 0.007918 | Limpopo |
| 65 | 0.259 | 0.189 | 1.148 | 0.014 | 0.000622 | 0.009865 | Limpopo |
| 66 | 0.215 | 0.275 | 1.440 | 0.011 | 0.000545 | 0.0106 | Limpopo |
| 67 | 0.096 | 0.336 | 1.888 | 0.011 | 0.000442 | 0.004567 | Limpopo |
| 68 | 0.404 | 3.467 | 13.767 | 0.003 | 0.001309 | 0.010256 | Limpopo |
| 69 | 0.046 | 0.247 | 0.821 | 0.002 | 0.000847 | 0.009032 | Limpopo |
| 70 | 0.145 | 0.421 | 2.704 | 0.004 | 0.000442 | 0.005001 | Free State |
